# Early Bone Formation around Immediately Loaded Transitional Implants Inserted in the Human Posterior Maxilla: The Effects of Fixture Design and Surface

**DOI:** 10.1155/2017/4152506

**Published:** 2017-02-09

**Authors:** Carlo Mangano, Jamil Awad Shibli, Jefferson Trabach Pires, Giuseppe Luongo, Adriano Piattelli, Giovanna Iezzi

**Affiliations:** ^1^Department of Dental Sciences, Vita Salute S. Raffaele University, 20132 Milan, Italy; ^2^Department of Periodontology and Oral Implantology, Dental Research Division, Guarulhos University, 07023070 Guarulhos, SP, Brazil; ^3^Department of Oral and Maxillofacial Surgery, Federico II University, 80183 Naples, Italy; ^4^Department of Medical, Oral and Biotechnological Sciences, G. d'Annunzio University, 66100 Chieti, Italy

## Abstract

*Aim*. To evaluate the effects of fixture design and surface on the early bone formation around immediately loaded implants inserted in the human posterior maxilla.* Materials and Methods*. Ten totally edentulous subjects received two transitional implants: one tapered implant with knife-edge threads/nanostructured calcium-incorporated surface (test: Anyridge®, Megagen, Gyeongbuk, South Korea) and one cylindrical implant with self-tapping threads/sandblasted surface (control: EZPlus®, Megagen). The implants were placed according to a split-mouth design and immediately loaded to support an interim complete denture; after 8 weeks, they were removed for histologic/histomorphometric analysis. The bone-to-implant contact (BIC%) and the bone density (BD%) were calculated. The Wilcoxon test was used to evaluate the differences.* Results*. With test implants, a mean BIC% and BD% of 35.9 (±9.1) and 31.8 (±7.5) were found. With control implants, a mean BIC% and BD% of 29.9 (±7.6) and 32.5 (±3.9) were found. The mean BIC% was higher with test implants, but this difference was not significant (*p* = 0.16). Similar BD% were found in the two groups (*p* = 0.9).* Conclusions*. In the posterior maxilla, under immediate loading conditions, implants with a knife-edge thread design/nanostructured calcium-incorporated surface seem to increase the peri-implant endosseous healing properties, when compared to implants with self-tapping thread design/sandblasted surface.

## 1. Introduction

In the last few years, the world of oral implantology and osseointegration has changed radically [1, 2].

In fact, new surgical techniques have been proposed, such as the placement of implants in extraction sockets [3, 4] and new prosthetic protocols, such as immediate [5, 6] or early [7] loading of the implants. These changes have been introduced to meet the modern needs of the patients, who wish to reduce the number of surgical sessions (and consequently the stress of the surgery and postoperative discomfort) and who want to be able to shorten the time of implant and prosthetic treatment [2, 3, 5].

The shortening of the treatment time translates into a reduction of the costs, with additional benefits for the clinician [4, 5].

However, the introduction of these new surgical and prosthetic protocols should not reduce, in the short and long term, the high percentages of survival and success recorded for rehabilitations supported by implants placed using conventional techniques, in fully healed ridges [8] and with delayed prosthetic loading [9, 10]. In fact, an increase in failures could be unacceptable for patients, who are increasingly demanding and would represent a major problem for clinicians [2, 4–6].

To be able to adapt to these new challenging surgical and prosthetic protocols, which continue to spread, while maintaining the high percentages of survival and success obtainable with conventional techniques, the industry has proposed a number of modifications and improvements of the implant macro- and microtopography [11–13].

The macrotopography (implant design) represents a very important element: it is believed that it can contribute significantly to the primary implant stabilisation [10, 11], together with patient-related factors (medical condition, bone quantity, and quality) [14, 15] and the experience and skills of the surgeon [16].

In fact, for the success of the implant therapy, it is well known that the fixture must have adequate stability at the time of positioning [5, 6, 11, 14]. In the absence of such stability, the risk of a failure is particularly high [11, 14]. The primary implant stabilisation is mainly of a mechanical nature, as it is determined mechanically by the interlocking between the threads of the implant and the preexisting bone at the recipient site [11, 14].

This primary stabilisation, however, must be followed by a proper secondary and biological stabilisation, due to the deposition, as fast as possible, of new bone onto the implant surface [7, 12, 13].

In fact, without this there is again the risk of implant failure due to a lack of osseointegration [12, 13]. Histologic studies have provided evidence that there is a period of bone remodeling following implant placement that results in a transient decrease in implant stability [17, 18]; resonance frequency analysis (RFA) evaluation has confirmed this evidence, reporting a drop in implant stability quotient (ISQ) values from the first to the third/fourth week following implant placement [19–21]. This reduction of the primary stability must therefore be balanced by an appropriate secondary stabilisation, determined by the deposition of new bone on the surface [11, 14, 22].

The influence of the macro- and micro/nanostructure of the implant on the success of osseointegration and in particular on the first healing phases of bone is now a subject of great interest for both researchers and clinicians [22]; the best way to assess the influence of design and implant surface on bone healing is certainly the histological and histomorphometric analysis of the interface between bone and implant [23, 24].

However, few studies to date have compared the influence of the macro- and micro/nanostructure of different implant systems on bone healing in humans [23, 25–29]: this is because it is difficult to perform comparative histologic and histomorphometric studies in humans, for ethical reasons.

Most of the studies available are based on a few samples [25] and implants are not subjected to immediate loading [23, 26, 27].

The purpose of this histological and histomorphometric study on humans is therefore to evaluate the early bone healing following the placement of implants with different macro- and microstructural characteristics, when positioned in the posterior maxilla and subjected to immediate loading.

## 2. Materials and Methods

### 2.1. Study Design

The present study was designed as a randomised controlled histologic/histomorphometric investigation, reporting on immediately loaded transitional transmucosal implants that were placed in the human posterior maxilla, and retrieved after a period of 8 weeks. In particular, this study aimed to compare the early bone response to tapered implants with knife-edge threads and a nanostructured calcium-incorporated surface (*test*: Anyridge, MegaGen, Gyeongbuk, South Korea) with the bone response to cylindrical implants with self-tapping threads and a sandblasted surface (*control*: EZPlus, MegaGen, Gyeongbuk, South Korea), when placed in the human posterior maxilla and subjected to immediate loading protocol. During a normal surgical procedure for the placement of conventional implants, each enrolled patient also received two transitional transmucosal implants (*n* = 1* test* implant; and *n* = 1* control* implant), which were inserted in the posterior maxilla, according to a split-mouth design. The transitional implants were placed with the aim of supporting an interim complete maxillary denture, until healing of the conventional implants. After 8 weeks, during the second-stage surgery to uncover the conventional implants, all transitional implants were retrieved for histologic/histomorphometric evaluation.

### 2.2. Patient Selection

A total of 10 fully edentulous patients (6 males, 4 females; aged between 46 and 77 years, mean age 61.7 ± 10.7, median 62, CI 95% 55.1–68.3) referred for oral rehabilitation with dental implants to the Oral Implantology Clinic, Dental Research Division, Guarulhos University, SP, Brazil, were consequently enrolled in the present study. The inclusion criteria were good systemic and oral health and sufficient native bone to place implants of 3.0 mm diameter and 6 mm length. The exclusion criteria were pregnancy, nursing, smoking, and any systemic condition that could affect bone healing. All participants received detailed explanations about the nature of the study and signed a written informed consent form. The Institutional Clinical Research Ethics Committee of Guarulhos University (CEP #201/03) approved the protocol of the present study, which was conducted in accordance with the Declaration of Helsinki on experimentation involving human subjects (2008).

### 2.3. Transitional Transmucosal Implants

The transitional transmucosal implants used in the present study were made of titanium grade 4. All implants were one-piece, 3.0 mm diameter × 6 mm length, but different in the macro- and micro/nanotopography.

**Figure 1:**
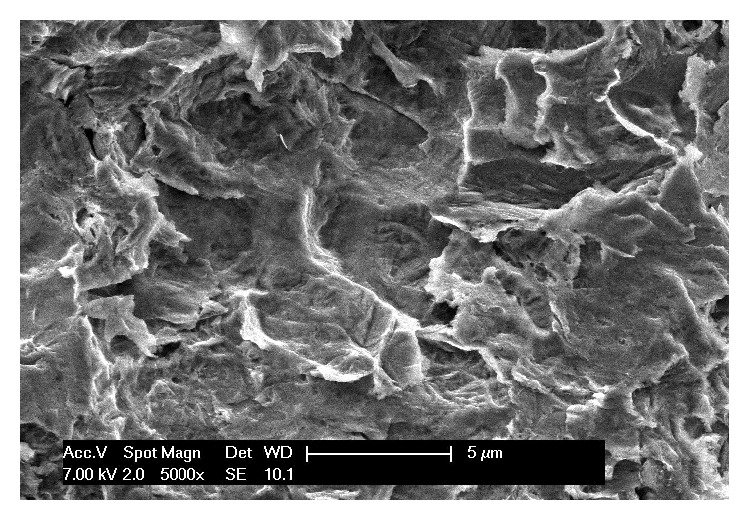
*Control* implant. Scanning electron microscopy of the resorbable blast media surface. Scanning electron microscopy evaluation revealed a mean *R*_*a*_ of 1.56 (±0.08) *μ*m, a mean *R*_*q*_ of 2.11 (±0.13) *μ*m, and a mean *R*_*t*_ of 18.53 (±1.56) *μ*m, respectively. Magnification 5000x.

The* control* implants (EZPlus, MegaGen, Gyeongbuk, South Korea) were cylindrical and featured a classical macroscopic design with self-tapping threads [22]. These implants were characterised by a surface blasted with particles of resorbable calcium phosphate (resorbable blast media, RBM). The surface was studied with scanning electron microscopy (SEM) (Figure 1) and the following standard roughness parameters were analysed: *R*_*a*_ (the arithmetic mean of the absolute height of all points), *R*_*q*_ (the square root of the sum of the squared mean difference of all points), and *R*_*t*_ (the difference between the highest and the lowest points). The scanning electron microscopy evaluation revealed a mean *R*_*a*_ of 1.56 (±0.08) *μ*m, a mean *R*_*q*_ of 2.11 (±0.13) *μ*m, and a mean *R*_*t*_ of 18.53 (±1.56) *μ*m, respectively.

**Figure 2:**
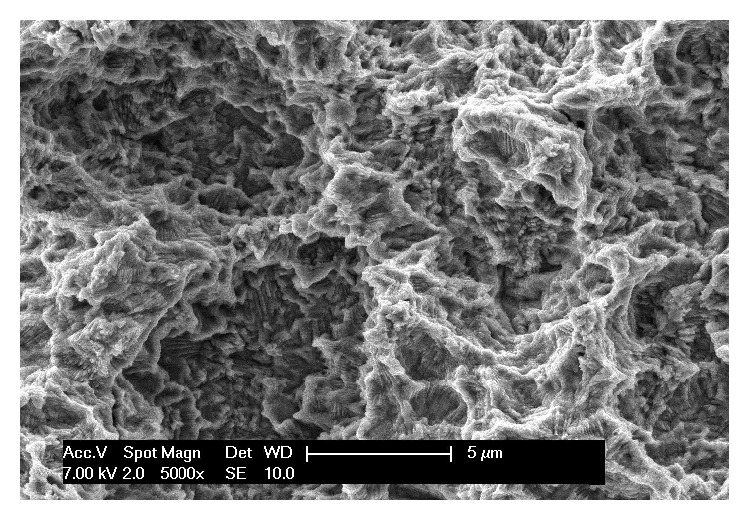
*Test* implant. Scanning electron microscopy of the nanostructured calcium-incorporated surface. Scanning electron microscopy evaluation revealed a mean *R*_*a*_ of 1.63 (±0.22) *μ*m, a mean *R*_*q*_ of 2.16 (±0.30) *μ*m, and a mean *R*_*t*_ of 15.76 (±0.29) *μ*m, respectively. Magnification 5000x.

Conversely, the* test* implants (Anyridge, MegaGen, Gyeongbuk, South Korea) were characterised by a tapered design with knife-edge, thin self-cutting threads [6, 22, 30–32]. The* test* implants had a nanostructured, calcium-incorporated surface (Xpeed®, Megagen Implant Co., Gyeongbuk, South Korea). This surface was obtained by modifying the original grit-blasted surface (resorbable blast media, RBM), which was enriched with calcium using a hydrothermal method. In brief, RBM implants were immersed in a mixed solution of 0.2 M sodium hydroxide (NaOH) and 2 mM calcium oxide (CaO) dissolved in deionized water using a Teflon-lined hydrothermal reactor system at 180°C for 24 h under a water pressure of 1 MPa^2^. With this procedure, a nanolayer of Ca^2+^ ions was incorporated onto the RBM surface, giving a CaTiO_3_ nanostructure [7, 30, 33]. Again, the surface was studied with scanning electron microscopy (SEM) (Figure 2). In this case, the SEM evaluation revealed a mean *R*_*a*_ of 1.63 (±0.22) *μ*m, a mean *R*_*q*_ of 2.16 (±0.30) *μ*m, and a mean *R*_*t*_ of 15.76 (±0.29) *μ*m, respectively.

### 2.4. Surgical Protocol

Twenty transmucosal transitional implants (*n* = 10* test* implants and *n* = 10* control* implants) were inserted in this study. All implants were placed under aseptic conditions. After local anaesthesia, a crestal incision connected with two releasing vertical incisions was made. Mucoperiosteal flaps were raised and conventional implants were inserted, in accordance with the surgical and prosthetic plan prepared for each patient. After placement of the conventional implants, two transitional transmucosal implants (*n* = 1* test* implant and *n* = 1* control* implant) were inserted in each patient, according to a split-mouth design. The transitional implants were inserted in the posterior region of the maxilla, among the conventional placed implants. The assignment of* test* and* control* implants (right posterior maxilla or left posterior maxilla) was random, as determined by a coin toss. The implant sites were prepared according to the manufacturer's recommendations, under profuse irrigation with sterile saline. The stability of all the implants was checked using a dedicated instrument (Osstell Mentor®, Osstell, Goteborg, Sweden): if an implant showed insufficient primary stability (implant stability quotient- ISQ <35), it was removed and a backup surgical site had to be prepared. The flaps were then sutured, to allow the emergency of the solid abutment of one-piece implants through the mucosa: these implants helped to support the interim maxillary denture during the entire healing period. Immediately after implant surgery, an interim maxillary denture was seated in the patient's mouth and relined intraorally. The stability of the interim complete denture, its retention, and the occlusion were carefully controlled. Clindamycin 300 mg (ClindaminC®, Teuto, Anapolis, Goias, Brazil) was administered three times a day for one week, to prevent infection. Postoperative pain was controlled with 600 mg ibuprofen (Actron®, Bayer Schering Pharma, Berlin, Germany) every 12 h for 2 days. To enable subjects to control postoperative dental biofilm, 0.12% chlorhexidine mouth rinses (Chlorhexidine®; Oral B, Boston, MA, USA) were prescribed, twice a day for 2 weeks. The sutures were removed after 10 days.

### 2.5. Specimen Retrieval and Histologic/Histomorphometric Analysis

The interim prosthesis remained connected to the transitional implants for a period of 8 weeks. After this period, during the 2-stage surgery to uncover the conventional implants, all clinically stable transitional fixtures (one* test* and* one* control implants) and the surrounding tissues were retrieved from each patient, using a 4.5-millimeter-wide trephine bur. Clinically mobile temporary implants were not considered for the histologic/histomorphometric evaluation. The specimens were fixed by immediate immersion at 10% buffered formalin and processed (Precise 1 Automated System®, Assing, Rome, Italy) to obtain thin sections, as previously described [23]. The specimens were dehydrated in an ascending series of alcohol rinses and embedded in glycol methacrylate resin (Technovit 7200 VLC®, Kulzer, Wehrheim, Germany). After polymerization, the specimens were cut longitudinally along the major axis of the implants with a high-precision diamond disc at about 150 *μ*m and ground down to about 30 *μ*m. Two slides were obtained for each implant. The slides were stained with basic fuchsin and toluidine blue. The specimens were studied using a transmitted-light microscope (Laborlux S®, Leitz, Wetzlar, Germany) interfaced with a high-resolution camera (3CCD-JVC KY-F55B®, JVC, Yokohama, Japan) and to a monitor and a personal computer (Intel Pentium III 1200 MMX®, Intel, Santa Clara, CA, USA). The whole system was connected to a digitizing pad (D-Pad®, Matrix Vision GmbH, Oppenweiler, Germany) and controlled by specific software for image capture (Image-Pro Plus® 4.5, Media Cybernetics, Immagini & Computer snc, Milan, Italy). For the histomorphometric evaluation, the bone-to-implant contact (BIC%), defined as the amount of mineralized bone in direct contact with the implant surface, was measured around all implant surfaces. Finally, the bone density (BD%) in a 500 *μ*m wide zone lateral to the implant surface was measured bilaterally, as previously reported.

### 2.6. Statistical Analysis

The mean, standard deviation, median, and confidence intervals (CI 95%) of histomorphometric values (BIC%, BD%) were calculated for each implant and then for each group of implants (*test* versus* control* implants). Comparisons of the differences in bone-implant percentages values in both groups were carried out using the Wilcoxon matched-pairs signed-rank test. The level of significance was set at 0.05. Results were presented as mean ± standard deviation (SD) and differences at *p* < 0.05 were considered statistically significant. All computations were carried out with a statistical analysis software (SPSS 17.0®, SPSS Inc., Chicago, IL, USA).

## 3. Results

### 3.1. Clinical Observations

Two months after placement, a total of 20 transitional transmucosal implants (*n* = 10* test* implants and *n* = 10* control *implants) were evaluated and retrieved. Two implants (one* test *implant and one* control* implant, placed in the same patient) were clinically unstable and showed no osseointegration, although they did not show any sign of infection. These two implants were excluded from the study and were not histologically/histomorphometrically evaluated. The remaining 18 implants were clinically stable at the time of retrieval and were therefore histologically/histomorphometrically evaluated.

### 3.2. Histologic/Histomorphometric Evaluation

**Figure 3:**
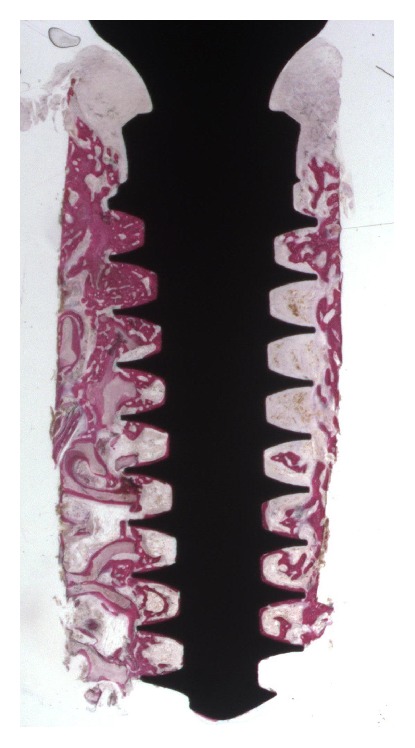
*Test* implant. Newly formed trabecular bone surrounded the whole implant perimeter. (Acid fuchsin and toluidine blue, magnification 12x).

**Figure 4:**
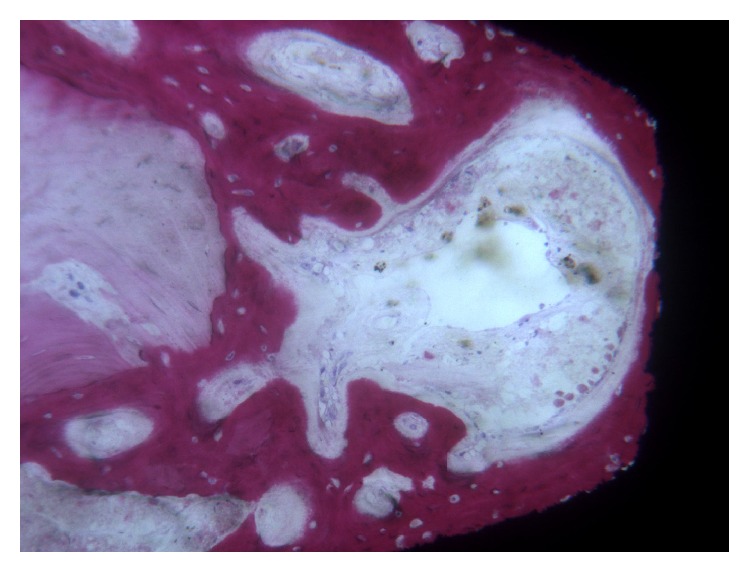
*Test* implant. The implant thread was lined by newly formed bone and an intense osteoblastic activity was still evident. (Acid fuchsin and toluidine blue, magnification 100x).

In the* test *implants, at low-power magnification, it was possible to see newly formed bone around and in contact with the implant surface. Around the implant collar, soft tissues were present. In the coronal portion, only newly formed bone with a trabecular structure and strongly stained with acid fuchsin could be observed. In the middle and apical portion of the implant, the native bone was evident far from the surface (Figure 3). At higher magnification, in the interthread concavities the newly formed bone was in contact with the implant surface and adapted perfectly to its microirregularities. Native bone in contact with newly formed bone could be seen. Osteoblasts secreting osteoid matrix near the bone-implant interface were found (Figure 4). Wide osteocyte lacunae could be observed often when they were in close vicinity to the implant surface. No inflammatory cell infiltrate was present. The histomorphometric evaluation revealed a BIC% of 35.9 ± 9.1 and a BD% of 31.8 ± 7.5, respectively. The BIC% ranged from 19.2 to 49.9; the median was 38.8; confidence interval (95%) was 29.9–41.8. The BD% ranged from 19.0 to 44.7; the median was 32.4; confidence interval (95%) was 26.9–36.7.

**Figure 5:**
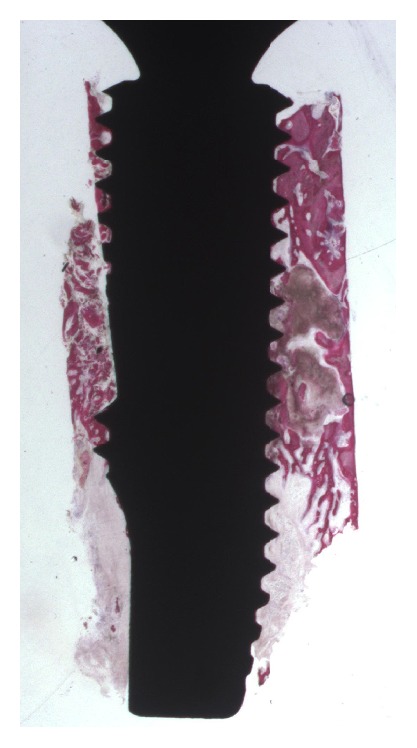
*Control *implant. The density of the bone tissue was different along the implant perimeter ranging from a more compact bone in the coronal portion to a very trabecular bone in the apical areas. (Acid fuchsin and toluidine blue, magnification 12x).

**Figure 6:**
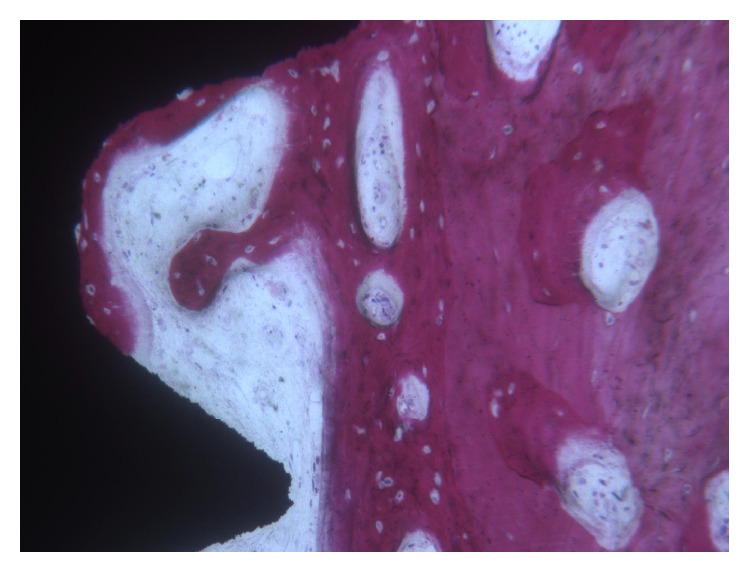
*Control *implant. Part of the implant thread was surrounded by newly formed bone and not yet mineralized osteoid matrix. (Acid fuchsin and toluidine blue, magnification 100x).

In the* control *implants, at low-power magnification, trabecular bone with small marrow spaces was mainly present in the coronal portion of the implant, while in the middle portion they tended to be wider (Figure 5). In the apical area, bone tissue was lacking. At higher magnification, newly formed bone tissue could be observed inside the thread concavity with osteocyte lacunae in contact with the surface. Not yet mineralized osteoid matrix could also be seen (Figure 6). The histomorphometric analysis revealed a BIC% of 29.9 ± 7.6 and a BD% of 32.5 ± 3.9, respectively. The BIC% ranged from 20.7 to 35.6; the median was 28.7; confidence interval (95%) was 24.6–35.2. The BD% ranged from 29.0 to 41.1; the median was 32.0; confidence interval (95%) was 29.8–35.2.

**Figure 7:**
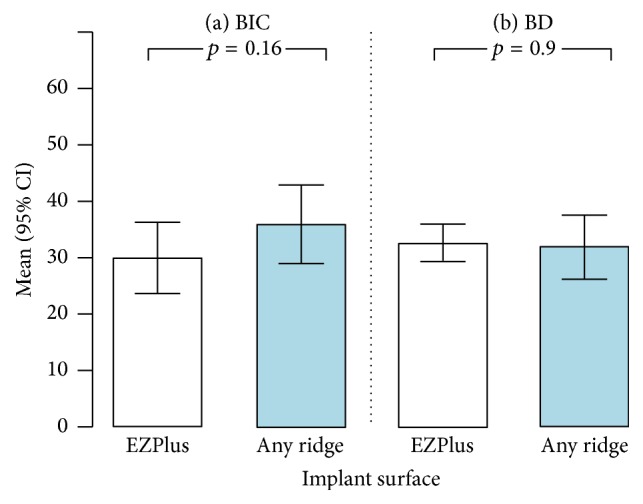
Histomorphometric results with EZPlus and Anyridge implants: bone-to-implant contact (BIC%) and bone density (BD%). In the EZPlus implants, the histomorphometric evaluation revealed mean (±SD) BIC% and BD% of 29.9 (±7.6) and 32.5 (±3.9), respectively. In the Anyridge implants, the histomorphometric analysis revealed mean (±SD) BIC% and BD% of 35.9 (±9.1) and 31.8 (±7.5), respectively.

Although the mean BIC% was higher in the* test* implants, this difference was not statistically significant (*p* = 0.16). Similar BD% were found in the two groups (*p* = 0.9). The histomorphometric results were summarised in Table 1 and displayed in Figure 7.

## 4. Discussion

At present, histologic/histomorphometric assessment is the most accurate method to investigate the bone healing processes and morphological characteristics of the bone-implant interface [22–24].

Unfortunately, only a few studies in the present literature have dealt with histologic/histomorphometric evaluation of human-retrieved implants [23, 25–29]; this is because of ethical issues related to implant retrieval from human subjects [23]. For this reason, little is known about the effects of different implant designs and surfaces on the early bone healing around dental implants [25].

In a recent systematic review reporting on human histologic/histomorphometric studies, the authors found that the bone-to-implant contact (BIC%) in the lower jaw is higher than in the upper jaw and that the BIC% in the anterior areas is higher than in the posterior areas [25]. In addition, they found that the implant design is a factor capable of affecting the BIC% [25]. In fact, the insertion of mini-implants in the posterior region results in lower outcomes, and differences were detected in the BIC% of standard length/diameter implants and mini-implants [25]. Finally, with regard to the loading protocols, the authors found that conventionally loaded implants had a higher BIC% than immediately loaded implants [25].

In the present randomised and controlled histologic/histomorphometric study, we have assessed the early bone healing of two different implants, under immediate loading in the human posterior maxilla. In particular, we have compared two different implants, with different design and surface, in order to understand which of the two could determine the best histologic and histomorphometric result. Twenty transitional transmucosal fixtures (6 mm length × 3.0 mm diameter) were inserted in the posterior maxilla, 10* test* implants and 10* control* implants; all these implants were subjected to immediate loading (as they helped to stabilise an interim complete removable denture) and remained in place for a period of two months, after which they were removed and analysed histologically.* Control* implants were characterised by a conventional macroscopic design, as well as by V-shaped, self-tapping threads, with four cutting edges; the surface of these implants was blasted with calcium phosphate particles (resorbable blast media treatment) and therefore it possessed microtopographic features.* Test *implants featured a novel knife-edge thread design. The surface of the* test *implants represented the development of the previous sandblasted surface, as a result of an ultrastructural treatment for superimposition/incorporation of calcium ions: it was therefore a nanostructured surface. Two months after placement and functional loading, the histologic evaluation revealed newly formed bone around and in contact with the surface of both implants; the new bone was formed in the interthread cavities, with osteoblasts secreting osteoid matrix near the bone-implant interface. These positive histologic outcomes were confirmed by the histomorphometric evaluation, with high percentages of bone-to-implant contact with both* test* and* control* implants. The histomorphometric results seemed to favour the* test* implants, for which a mean value (±SD) of BIC% corresponding to 35.9% (±9.1) was obtained; this value was higher than that found in* control *implants, which corresponded to 29.9 (±7.6). The contact between bone and implant values was higher in* test* implants; however, this difference was not statistically significant (*p* = 0.16). The BD% values were instead equivalent in the two groups (*p* = 0.9), with an average value for the* test* fixtures (31.8 ± 7.5) which was similar to that reported for the* control* fixtures (32.5 ± 3.9).

In the present study, the BIC% of the* test* implants (35.9% ± 9.1) was higher than that of the* control* implants (29.9 ± 7.6), although there was no statistically significant difference (*p* = 0.16) between the two groups. This result is not negligible. In fact, in particularly difficult clinical contexts such as the placement of implants in low quality bone areas (posterior maxilla) [6, 7, 10, 18] or in the case of immediate loading protocols [1, 2, 4, 6], it is important to achieve and maintain, in the short and medium term, high percentages of contact between bone and implant. This is because, in the end, high percentages of contact between bone and implant can determine the success, or failure, of the therapy [22].

At the time of positioning, the implant stabilisation is obtained mechanically, through the interlocking between the implant threads and the preexisting bone [10, 11, 14, 22]; however, in the next 3-4 weeks, a partial resorption of the bone tissue involved in this primary stabilisation occurs physiologically [11, 14, 22]. It is therefore necessary to deposit new bone on the implant surface, to counteract this physiological resorption and to avoid the mobilisation (and loss) of the implant [14, 22].

The aim of modern implantology is therefore twofold: on the one hand, it aims to maximise the primary stability at implant placement, through the search for new designs and macrotopographies that enable effective stabilisation and a high bone-to-implant contact [22, 34, 35]; on the other hand, it intends to counteract the physiological fall of stability occurring due to remodeling phenomena, stimulating new bone deposition on the implant, through the use of bioactive surfaces [12, 13, 33, 36].

In the present work, the best result of contact between bone and implant can be due either to the design or the surface of the* test* implants. The novel thread design of* test* implants may, in fact, result in maximum bone-to-implant contact (BIC), maximised compressive force resistance, and minimised shear force production; thereby it has the potential to prevent a drop in stability in the immediate postplacement healing period [22, 34]. At the same time, the novel nanostructured calcium-incorporated surface of* test *implants may stimulate a faster new bone formation onto the implant surface, through increased surface area and increased free energy, as currently reported in the scientific literature [7, 12, 13, 33, 35, 36].

The present histologic/histomorphometric study supports the concept that implants with knife-edge threads and a nanostructured calcium-incorporated surface seem to represent the best choice in the event of clinically challenging situations (such as areas of poor bone quality, or immediate loading protocols), at least when compared with implants with self-tapping threads and a sandblasted surface. Several clinical studies have confirmed that implants with knife-edge threads and a nanostructured calcium-incorporated surface can successfully support different kinds of prosthetic restorations, under different loading protocols, with high survival rates, at least in the short term [6, 7, 30–32, 37].

The present study has limitations. A critical factor for the present study is the fact that we have compared two implant systems that are characterised by different designs (macrotopography) and surfaces (micro/nanotopography); to better assess the effects of micro/nanotopography of the implant surface on early bone healing, it would be more appropriate to compare two macroscopically identical fixtures that differ only in the surface [23, 26–28]. Similarly, to more effectively assess the effects of macrotopography on early bone healing, it would have been more appropriate to compare implants with different thread designs, but characterised by the same surface topography. Another limitation of this study is the number of enrolled patients (10) and positioned implants (20): a larger number of patients and implants would certainly have been preferable, but in the specific case it was not possible to enrol more than 10 patients. Moreover, in the present work, two fixtures (1* test* implant and 1* control *implant, inserted in the same patient) were not clinically stable at the time of removal, due to lack of osseointegration: these fixtures were excluded from histologic and histomorphometric evaluation, and this could be another limitation of our research. Finally, in the present study we have used implants of reduced dimensions (6.0 mm in height × 3.0 mm in diameter): this may be a limitation because the use of standard length and diameter implants could lead to different results, compared to the outcomes found here. If we examine it more carefully, however, the fact that the small implants have been used may even be an advantage of this study: in fact, excellent histologic and histomorphometric results have emerged from the removal, after 2 months of functional loading, of these immediately loaded, short, and narrow fixtures [27, 28], placed in the posterior maxilla. In any case, it would not have been ethically possible here to use implants with a standard length and diameter. Further randomised controlled studies on a larger number of patients are required, in order to confirm the positive findings from this work.

## 5. Conclusions

In the present histologic/histomorphometric study in the human posterior maxilla, immediately loaded implants with a knife-edge thread design and nanostructured calcium-incorporated surface increased the peri-implant endosseous healing properties, when compared with immediately loaded implants with a self-tapping thread design and sandblasted surface. The present data must be considered with caution because of the study design and methodology (only stable implants were evaluated) and the limited number of patients enrolled and fixtures inserted. Therefore, additional controlled randomised clinical studies are needed to draw more specific conclusions about the early bone response to implants with a knife-edge thread design and a nanostructured calcium-incorporated surface.

## Figures and Tables

**Table 1:** Bone to implant contact (BIC%) and bone density (BD%): means, standard deviations, medians, ranges, and confidence intervals for *test *and *control *implants, respectively.

	Mean	SD	Median	Range	CI 95%	*p*
BIC%						
*Test implants*	35.9	9.1	38.8	19.2–49.9	29.9–41.8	0.16
*Control implants*	29.9	7.6	28.7	20.7–35.6	24.6–35.2	
BD%						
*Test implants*	31.8	7.5	32.4	19.0–44.7	26.9–36.7	0.9
*Control implants*	32.5	3.9	32.0	29.0–41.1	29.8–35.2	
